# Educational

**Published:** 1871-04

**Authors:** 


### EDUCATIONAL.

The closing exercises of the Twenty-fifth Annual Session of the Ohio College of Dental Surgery, took place on the evening of March 1st. The commencement was held in the hall of the college. As usual, upon such occasions, the hall was filled with an interested audience. The senior class consisted of nine members, viz.: J. A. Stipp, Ohio: Chas. Welch, Ohio; John Rabe, Ohio; R. E. Finley, Ohio; W. T. Wallace, Ohio; S. E. Harryman, Indiana: J. E. Cravens, Indiana; J. R. Wilkings, Mississippi; C. S. Case, Michigan, upon each of whom was conferred the degree of Doctor of Dental Surgery, by Dr. James Taylor, President of the Board of Trustees, who also delivered an interesting address. The valedictory to the graduating class was delivered by Dr. F. H. Rehwinkle, of Chillicothe, O., for which see another page of this journal, it speaks for itself. We ask for it a careful perusal, and some parts of it should be committed to memory. It should be brought to the notice of the public as well as the profession.

The reply on behalf of the class was made by J. E. Cravens, and is just what it should be. In addition, a fine band discoursed sweet music during the evening, and to all it was a very pleasant conclusion of the arduous labors of the session. T.

